# Prevalence and Accuracy of Information on CYP2D6, CYP2C19, and CYP2C9 Related Substrate and Inhibitor Co-Prescriptions in the General Population: A Cross‐Sectional Descriptive Study as Part of the PharmLines Initiative

**DOI:** 10.3389/fphar.2020.00624

**Published:** 2020-05-08

**Authors:** Muh. Akbar Bahar, Jens H. J. Bos, Sander D. Borgsteede, Aafje Dotinga, Rolinde A. Alingh, Bob Wilffert, Eelko Hak

**Affiliations:** ^1^Unit of PharmacoTherapy, -Epidemiology & -Economics, Groningen Research Institute of Pharmacy, University of Groningen, Groningen, Netherlands; ^2^Faculty of Pharmacy, Hasanuddin University, Makassar, Indonesia; ^3^Department of Clinical Decision Support, Health Base Foundation, Utrecht, Netherlands; ^4^Lifelines Cohort Study, Lifelines Databeheer B.V., Roden, Netherlands; ^5^Department of Clinical Pharmacy and Pharmacology, University Medical Center Groningen, Groningen, Netherlands

**Keywords:** CYP2D6, CYP2C19, CYP2C9, drug-drug-interaction, Lifelines, IADB.nl

## Abstract

**Background:**

Drug-drug interaction (DDI) is one of the main contributors to adverse drug reactions and therefore, it is important to study its frequency in the population. We aimed to investigate frequency and concordance on CYP2D6, CYP2C19, and CYP2C9 (CYP2D6/2C19/2C9)-mediated potential DDIs at the Lifelines cohort and linked data from the pharmacy database IADB.nl.

**Methods:**

As part of the University of Groningen PharmLines Initiative, data were collected on CYP2D6/2C19/2C9-related substrate/inhibitors from entry questionnaires of Lifelines participants and linked information from the pharmacy database IADB.nl. CYP2D6/2C19/2C9 related co-prescriptions were divided based on the type of drugs i.e. chronically used medication (CM) or occasionally used medication (OM). This resulted in the combination of two chronically used drugs (CM-CM), chronically and occasionally used medication (CM-OM), and two occasionally used drugs (OM-OM). To measure the agreement level, cohen’s kappa statistics and test characteristics were used. Results were stratified by time window, gender, and age.

**Results:**

Among 80,837 medicine users in the Lifelines, about 1–2 per hundred participants were exposed to a CYP2D6/2C19/2C9-mediated potential DDI. Overall, the overlapping time window of three months produced the highest mean kappa values between the databases i.e. 0.545 (95% CI:0.544–0.545), 0.512 (95% CI:0.511–0.512), and 0.374 (95% CI:0.373–0.375), respectively. CM-CM had a better level of agreement (good) than CM-OM (fair to moderate) and OM-OM combination (poor to moderate). The influence of gender on concordance values was different for different CYPs. Among older persons, agreement levels were higher than for the younger population.

**Conclusions:**

CYP2D6/2C19/2C9-mediated potential DDIs were frequent and concordance of data varied by time window, type of combination, sex and age. Subsequent studies should rather use a combination of self-reported and pharmacy database information.

## Introduction

Drug-drug interactions (DDIs) are an important contributor to adverse drug reaction leading to hospitalization or mortality (Doucet et al., 1996; Montane et al., 2018). CYP2D6, CYP2C19, and CYP2C9 (CYP2D6/2C19/2C9), subtypes of CYP450 drug metabolizing enzymes, are commonly involved in mediating partly inappropriate DDIs as these enzymes metabolize a wide variety of drugs in clinical practice (Flockhart and Oesterheld, 2000; Bahar et al., 2017b). CYP2D6/2C19/2C9 are highly polymorphic enzymes and the genetic polymorphisms produce inter-individual variabilities in drug metabolisms ranging from poor to accelerated metabolic activities (Zanger and Schwab, 2013). Consequently, the clinical impact of CYP2D6/2C19/2C9-mediated DDIs might be variable from person to person and depends on his/her genetic profile (Bahar et al., 2017a). The information on the clinical relevance and management of DDIs mediated by different CYP2D6/2C19/2C9 genotypes is therefore needed. In order to provide the information, the first step that needs to be done is to generate the data about the burden and type of potential DDIs mediated by these enzymes in the general population.

Estimation of the prevalence rate of a potential DDI is commonly performed using self-reporting methods in which patients are interviewed or filled out a self-administered questionnaire (Van den Brandt et al., 1991; Classen et al., 2007; Secoli et al., 2010). However, this kind of assessment is prone to information bias, because of inaccurate recall, which may influence the validity of results (Rockenbauer et al., 2001; West et al., 2005). Hence, it is important to validate drug information collected with self-reporting methods (Haapea et al., 2010; Hafferty et al., 2018).

The Lifelines cohort is a Dutch three-generation population cohort that provides a wide variety of medical and non-medical data, genomic information, and data on medication use (Stolk et al., 2008; Scholtens et al., 2014). The Lifelines cohort, as a prospective and long-term database, offers possibilities in pharmaco-epidemiological studies, such as assessing the impact of gene polymorphism on the magnitude of DDIs in the population. However, currently not much is known about the frequency, type and validity of potential DDIs in the open population.

This study has both a methodological and an epidemiological aim: we studied the frequency of potentially interacting substrates and inhibitors of the CYP2D6/2C19/2C9 and the concordance level of the information derived by self-reported drug use and an analysis of data from a drug-use database. For the latter aim, information as observed in the Lifelines cohort was compared with data from a prescription database, the University of Groningen prescription database IADB.nl, across type of medications, sex, and age (Visser et al., 2013; Sediq et al., 2018). A prescription database is regarded as an accurate database and not to be influenced by so-called recall bias (Monster et al., 2002; Schneeweiss and Avorn, 2005). Additionally, IADB.nl has been proven a reliable database in many pharmaco-epidemiological studies (Daud et al., 2017; Alfian et al., 2018; Bahar et al., 2018b).

## Materials and Methods

The PharmLines Initiative is a university wide project in which the data of the Lifelines Cohort study have been linked to the University of Groningen prescription database IADB.nl. The project was started in 2017 by the Groningen Research Institute of Pharmacy, Departments of Epidemiology and Clinical Pharmacy, Department of Pharmacology of the University Medical Center Groningen and the Lifelines Cohort Study (https://www.lifelines.nl/researcher/cohort-and-biobank) (Sediq et al., 2018).

### The Lifelines Cohort

The Lifelines cohort covers 167,729 participants from the Northern part of the Netherlands, aged 6 months until 93 years old, which were recruited from 2006 until 2013 (Stolk et al., 2008; Scholtens et al., 2014). It is an observational cohort study intended to facilitate research on the contribution and interaction between environmental, genetic, and phenotypic aspects in the development of chronic diseases and healthy aging (Stolk et al., 2008; Scholtens et al., 2014). The recruited participants will be followed for at least 30 years and are asked to complete a questionnaire every 1.5 years. In addition, once every 5 years, the participants have a comprehensive physical examination (Stolk et al., 2008; Scholtens et al., 2014). Baseline questionnaires included questions about general information, lifestyle and environment, psychosocial aspects, and health (including medication use) (Stolk et al., 2008; Scholtens et al., 2014; Klijs et al., 2015). The medication use information were collected in two ways i.e. a) patients filled out a questionnaire or b) patients carried the medication at the time of interview (Sediq et al., 2018). The medication data regarding their current prescription and dose were recorded and classified using the Anatomical Therapeutic Chemical (ATC) coding scheme (Stolk et al., 2008; Scholtens et al., 2014). The Lifelines population is multigenerational and generally representative of the Dutch population resided in the Northern part of the Netherlands (Klijs et al., 2015).

### University of Groningen IADB.nl Database

The University of Groningen prescription database IADB.nl has recorded prescriptions from community pharmacies in the Netherlands since 1994, and is updated annually (Visser et al., 2013; Sediq et al., 2018). In 2017, it contained prescription data of approximately 700,000 individuals from around 72 pharmacies that are located in most of the area where the Lifelines cohort is also resident. The study population was reported to represent the general population in Netherlands (Visser et al., 2013; Sediq et al., 2018). In the IADB.nl, each patient has a unique and anonymous identifier. Each record contains information about patient’s sex, date of birth, and information about his/her prescribed medication such as ATC code, duration, daily dose, amount prescribed, and dispensing date (Visser et al., 2013; Sediq et al., 2018). The IADB.nl has no information about over-the-counter (OTC) drugs and prescriptions from the hospital.

### Study Population and Linkage of Databases

The study population consists of all medicine users (≥18 years) in the Lifelines cohort. A Trusted Third Party, Statistics Netherlands (Dutch: *Centraal Bureau voor de Statistiek;* CBS), carried out the linkage of the Lifelines and the IADB.nl records at the patient level based on postal code in combination with sex and date of birth. The unique identifiers from both databases were removed, and once the linkage was completed, each patient was assigned a new unique code that cannot be traced back to their previous identifier. Using the new identifier, the data from both databases could be combined. The complete linking process was described in more detail by Sediq et al. (Sediq et al., 2018).

### Exposures

Exposures were defined as substrates and inhibitors of CYP2D6/2C19/2C9. We defined a potential DDI as each combination of a substrate and inhibitor listed in the international standard and local guideline, Flockhart Table for CYP-mediated drug interactions and *the Dutch Commentaren Medicatiebewaking book*, respectively (Borgsteede, 2015; Flockhart, 2018). Based on the main indication according to the official product information, the exposures were classified as: 1) chronically used medication (CM) for example CYP2D6 substrates such as beta-blockers (metoprolol), and 2) occasionally used medication (OM) for example CYP2D6 substrates such as opioids (tramadol). The full list of medications including their classification can be found in **Supplementary Material 1**.

### Outcomes

Outcome measures were defined as frequency of potential CYP2D6/2C19/2C9-mediated DDIs as well as the levels of agreement between the self-reported information from the Lifelines cohort and the IADB.nl prescription data on these potential DDIs across type of medications, age, and sex. If one patient was exposed to different types of CYP2C9/2D6/2C19 mediated DDIs, we calculated them as one participant with several incidences of potential DDIs. If the potential DDI was only found in the Lifelines cohort records, it was categorized as over-reporting (false positive). If the potential DDI was only found in the IADB.nl, it was categorized as under-reporting (false negative). We also provided data on sensitivity, specificity, negative predictive value/NPV, and positive predictive value/PPV for the top five potential DDIs detected in the lifelines database. Different overlapping time windows (i.e. 1 month, 3 months, 6 months, 9 months, and 1 year) between baseline date of self-reporting medication in the Lifelines cohort and dispensing date of prescription in the IADB.nl were applied to determine the optimum time window for assessing the agreement of both databases. Subgroup analyses by the type of medication (CM vs OM), age, and sex were performed to observe the potential influence of these factors on the agreement. Additionally, we also presented information about the clinical relevance of the potential DDIs based on the suggested management provided by Epocrates^®^ i.e. “contraindicated, avoid combination/use alternative, modify treatment/monitor and caution”. If Epocrates^®^ had no recommendation for the potential DDI, we checked whether Drugs.com, another online drug interactions screening software, provided suggestions for the potential DDI. Both of them were reported to have a high sensitivity for detection of potential DDIs (Perkins et al., 2006; Bossaer and Thomas, 2017).

### Statistical Methods

Comparisons of the prevalence of potential CYP2D6/2C19/2C9-mediated DDIs [mean (SD)] and the frequency of participants with the potential DDIs [number (%)] across age groups (18–59 vs >=60 years old) and sex (men vs women) were performed by using independent sample t-test. A p-value which is less than 0.05 (< 0.05) is considered to indicate a statistically significant difference between comparison groups. Multivariate analysis of the influence of age and sex on the risk of having the potential DDIs was conducted by using a binary logistic regression method to obtain the crude and covariate-adjusted odds ratios as a measure of association. A p-value < 0.05 and 95% confidence interval (CI) not including 1 are considered as indicators for significant associations. To determine the agreement values between the databases on the potential DDIs, we used Cohen’s kappa statistics and 95% CI. Altman et al. provided some guidelines to define the Cohen’s kappa values i.e. poor (< 0.20), fair (0.20–0.40), moderate (0.41–0.60), good (0.61–0.80), and very good (0.81–1.00) (Altman, 1990).

## Results

**Figure 1:**
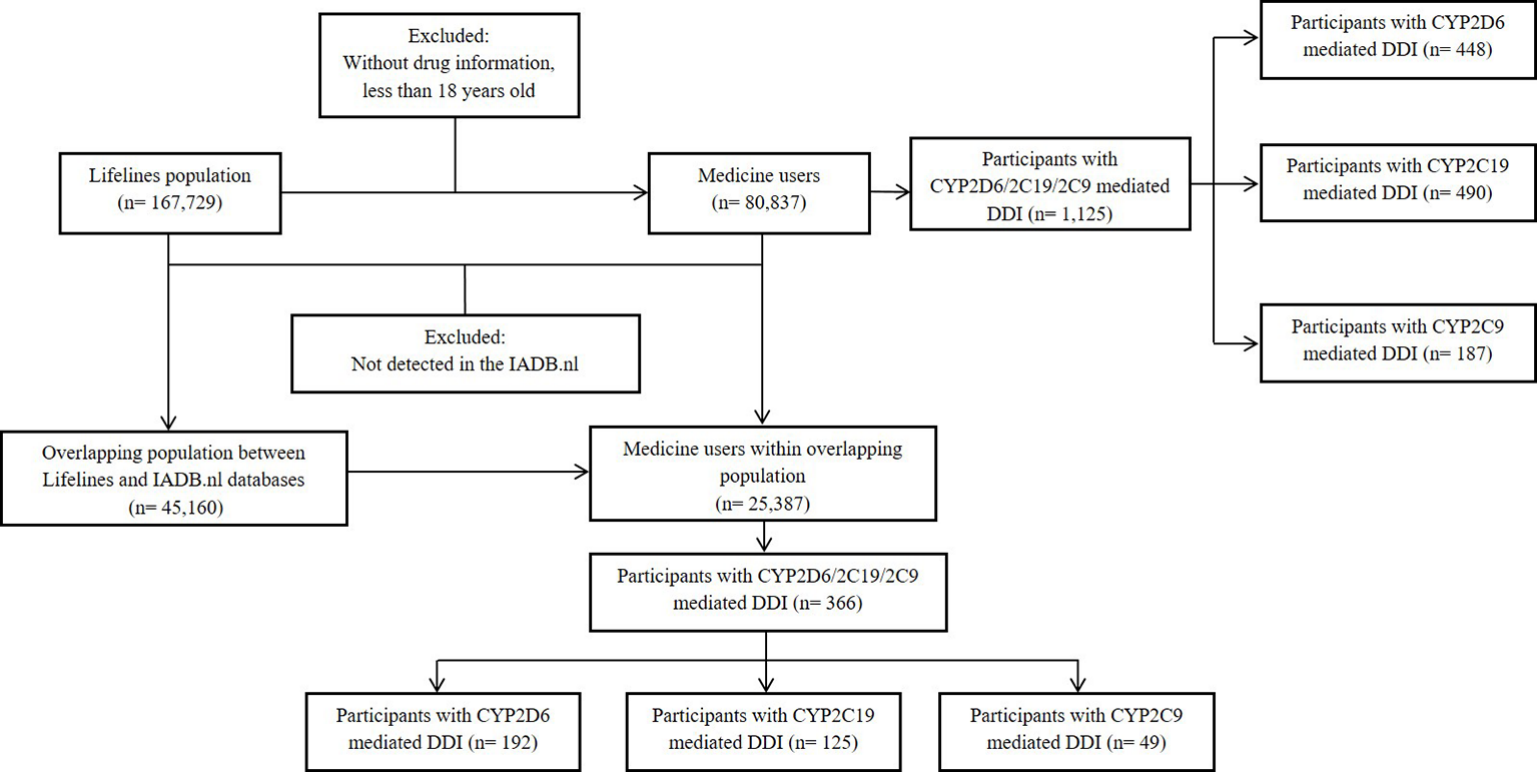
Selection of the population study. DDI, drug-drug interaction.

Among of 167,729 Lifelines participants, 80,837 adults were recorded with self-reported medicine use (mean age 46 years and 68.5% women) in the cohort at entry (**Table 1**). Among the subjects, there were 1,125 (1.4%) self-reported medicine users exposed to 1,199 potential CYP2D6/2C19/2C9-mediated DDIs (**Figure 1**). The prevalence of potential CYP2D6/2C19/2C9-mediated DDIs was 488, 513, and 198 respectively (**Table 2**). Older women had a significantly higher risk to be exposed to CYP2D6 (OR: 2.159, 95% CI: 1.386–3.363) and CYP2C19 (OR: 1.691, 95% CI: 1.184–2.416) mediated DDIs than older men but the comparable risks were observed among younger group. There was also a tendency that women had an increased risk to be exposed to CYP2C9 mediated DDIs than men (**Table 3**).

**Table 1:** Characteristics of participants with self-reported medication use at entry in the Lifelines cohort database and overlap with IADB.nl prescription database.

Characteristics	Number of participants (n = 80,837)
Age in year, mean (± SD)	46.13 (± 14.21)
18-59 years old, N (%)	64,807 (80.17%)
>= 60 years old, N (%)	16,030 (19.83%)
Gender, N women (%)	55,352 (68.50%)
Total participants with CYP2D6/2C19/2C9 mediated DDI, N(%)	1,125 (1.40%)
Total participants overlapped with IADB.nl database, N (%)	25,387 (31.41%)
Age in year, mean (± SD)	45.54 (± 14.62)
18-59 years old, N (%)	20,277 (79.90%)
>= 60 years old, N (%)	5,110 (20.10%)
Gender, N women (%)	17,416 (68.60%)
Total participants with CYP2D6/2C19/2C9 mediated DDI, N (%)	366 (1.44%)

SD, Standard Deviation; DDI, drug-drug interaction.

**Table 2:** Prevalence and participants with potential DDIs in the Lifelines cohort.

Variables	Prevalence of potential DDIs (*n* = 1,199)						Variables	Participants with potential DDIs (*n* = 1,125)					
	Age in years [mean (SD)]		P-value	Gender [mean (SD)]		P-value		Age in years [n (%)]		P-value	Gender [n (%)]		P-value
	18-59	>=60		Men	Women			18-59	>=60		Men	Women	
CYP2D6 (*n* = 488)	0.006 (0.09)	0.006 (0.01)	0.519	0.005 (0.08)	0.006 (0.08)	0.048	CYP2D6 (*n* = 448)	349 (0.54)	99 (0.62)	0.227	118 (0.46)	330 (0.59)	0.018
CYP2C19 (*n* = 513)	0.006 (0.08)	0.009 (0.09)	0.0002	0.006 (0.08)	0.007 (0.08)	0.428	CYP2C19 (*n* = 490)	351 (0.54)	139 (0.87)	0.000002	148 (0.58)	342 (0.62)	0.527
CYP2C9 (*n* = 198)	0.003 (0.05)	0.002 (0.05)	0.178	0.002 (0.04)	0.003 (0.05)	0.037	CYP2C9 (*n* = 187)	156 (0.24)	31 (0.19)	0.264	47 (0.18)	140 (0.25)	0.060

SD, Standard Deviation; DDI, drug-drug interaction.

**Table 3:** Multivariate analysis on the influence of age and sex on risk of having potential CYP2C9/2D6/2C19 mediated DDIs.

Multivariate analysis					Sub-group analysis		
Variables	Crude OR (95% CI)	P-Value	Adjusted OR (95% CI)	P-Value	Variable	OR (95% CI)	P-Value
*CYP2D6* (*n* = 448)							
Age					18–59		
18–59	Ref.		Ref.		Men	Ref.	
>=60	1.148 (0.918–1.436)	0.228	1.119 (0.956–1.503)	0.116	Women	1.119 (0.881–1.422)	0.358
Sex					>=60		
Men	Ref.		Ref.		Men	Ref.	
Women	1.289 (1.044–1.592)	0.018	1.319 (1.066–1.632)	0.011	Women	2.159 (1.386–3.363)	0.001
*CYP2C19 (n* = 490*)*							
Age					18–59		
18–59	Ref.		Ref.		Men	Ref.	
>=60	1.606 (1.319–1.956)	0.000002	1.640 (1.343–2.002)	0.000001	Women	0.949 (0.754–1.195)	0.659
Sex					>=60		
Men	Ref.		Ref.		Men	Ref.	
Women	1.064 (0.877–1.291)	0.528	1.139 (0.936–1.385)	0.194	Women	1.691 (1.184–2.416)	0.004
*CYP2C9 (n* = 187*)*							
Age					18–59		
18–59	Ref.		Ref.		Men	Ref.	
>=60	0.803 (0.546–1.181)	0.265	0.841 (0.570–1.241)	0.383	Women	1.316 (0.906–1.910)	0.149
Sex					>=60		
Men	Ref.		Ref.		Men	Ref.	
Women	1.372 (0.986–1.910)	0.061	1.345 (0.964–1.878)	0.081	Women	1.466 (0.702–3.063)	0.308

SD, Standard Deviation; DDI, drug-drug interaction; OR, Odds Ratio; CI, Confidence Interval.

**Figure 2:**
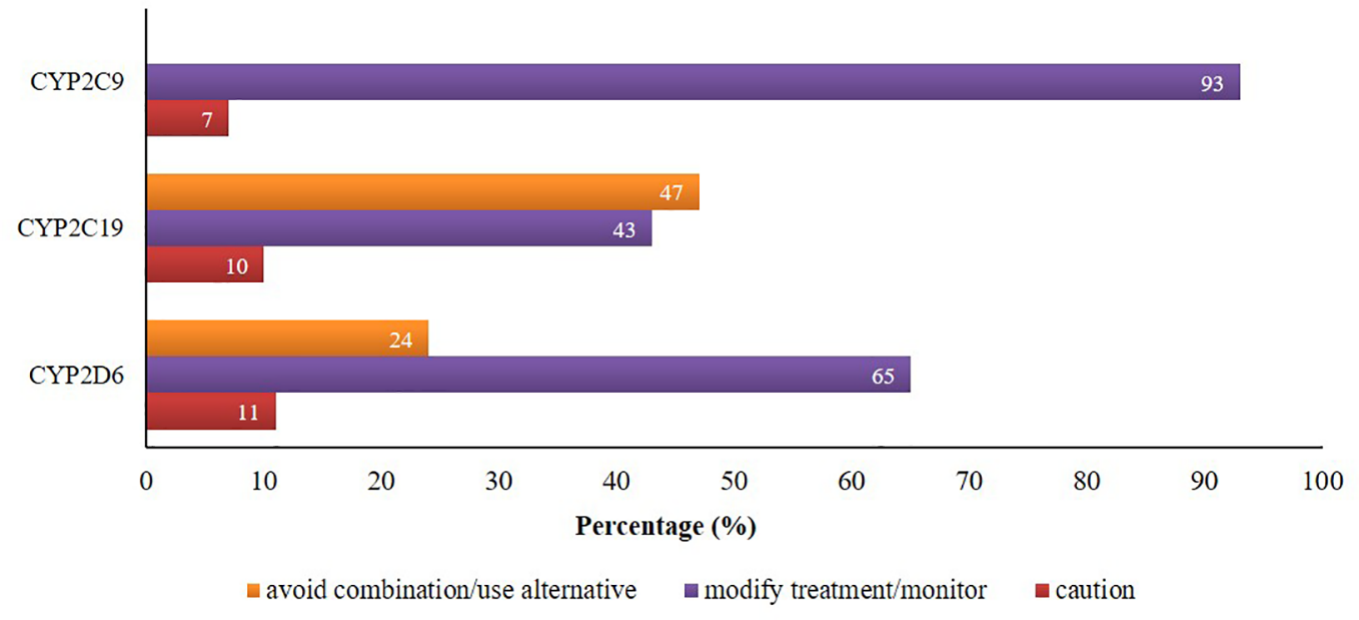
Proportion of potential DDIs based on the suggested managements provided by Epocrates® and Drugs.com.

There were 24% and 47% of CYP2D6 and CYP2C19 mediated co-prescriptions, respectively, which were in the category of “avoid combination/use alternative”. Additionally, about 65%, 43%, and 93% of CYP2D6/2C19/2C9-mediated combinations were in the category of “modify treatment/monitor” according to the knowledgebase (**Figure 2**).

Information from 45,160 Lifelines participants could be linked to the IADB.nl database. Among this linked population, there were 25,387 self-reported medicine users with comparable age and sex distribution (mean age 45.5 years and 68.6% women) as observed in the total medicine users in the Lifelines cohort (**Table 1**). Metoprolol-paroxetine (83 events), citalopram-omeprazole (173 events), and diclofenac-paroxetine (51 events) were the most prevalent potential DDIs mediated by CYP2D6/2C19/2C9, with good, moderate, and fair agreement of questionnaire and prescription data, respectively. Data on kappa, sensitivity, specificity, PPV and PPV values of the top five most frequent potential DDIs in the Lifelines database can be found in **Table 4**. Information on self-reported combinations of chronically used medications such as metoprolol-fluoxetine had very good agreement, high sensitivity and specificity as well as high PPV and NPV. Meanwhile, information on self-reported combinations with occasionally used medication such as ibuprofen-paroxetine tended to have fair kappa, sensitivity, and PPV but high specificity and NPV. The complete list of the potential DDIs in the Lifelines database and their kappa values can be found in the **Supplementary Material 2** and **3**, respectively.

**Table 4:** Top five potential DDIs in the Lifelines cohort detected in the IADB.nl database with their kappa, sensitivity, specificity, PPV, and NPV, as well as 95% CI (time window: 3 months).

CYP2D6/2C19/2C9 mediated potential DDI											
Potential DDI	N^a^	N1^b^	N2^c^	Detected in both databases (TP)	Over-reporting^d^ (FP)	Under-reporting^e^ (FN)	Kappa (95%CI)	Sensitivity, % (95%CI)	Specificity, % (95%CI)	PPV, % (95%CI)	NPV, % (95%CI)
*CYP2D6*											
metoprolol_paroxetine	83	39	28	22	17	6	0.656 (0.655–0.656)	78.57 (59.05–91.07)	99.93 (99.89–99.96)	56.41 (43.65–63.37)	99.98 (99.95–99.99)
metoprolol_clomipramine	18	15	11	10	5	1	0.769 (0.768–0.770)	90.91 (58.72–99.77)	99.98 (99.95–99.99)	66.67 (44.94–83.05)	100.00 (99.97–100.00)
metoprolol_fluoxetine	17	10	9	9	1	0	0.947 0.946–0.947)	100.00 (66.37–100.00)	99.99 (99.98–100.00)	90.00 (55.90–98.46)	100.00 (-)
metoprolol_amiodarone	14	11	5	5	6	0	0.625 (0.623–0.627)	100.00 (47.82–100.00)	99.98 (99.95–99.99)	45.45 (27.24–64.97)	100.00 (-)
metoprolol_duloxetine	14	3	2	2	1	0	0.800 (0.797–0.802)	100.00 (15.81–100.00)	100.00 (99.98–100)	66.67 (21.98–93.42)	100.00 (-)
*CYP2C19*											
citalopram_omeprazole	173	37	29	19	18	10	0.575 (0.574–0.576)	65.52 (45.67–82.06)	99.93 (99.89–99.96)	51.35 (38.27–64.25)	99.96 (99.93–99.98)
diazepam_omeprazole	151	40	49	19	21	30	0.425 (0.424–0.426)	38.78 (25.20–53.76)	99.92 (99.87–99.95)	47.50 (34.21–61.15)	99.88 (99.85–99.91)
omeprazole_fluvoxamine	28	5	4	3	2	1	0.667 (0.665–0.669)	75.00 (19.41–99.37)	99.99 (99.97–100.00)	60.00 (25.13–87.02)	100.00 (99.98–100.00)
diazepam_esomeprazole	27	8	10	5	3	5	0.555 (0.553–0.557)	50.00 (18.71–81.29)	99.99 (99.97–100.00)	62.50 (31.45–85.83)	99.98 (99.96–99.99)
clopidogrel_omeprazole	24	10	8	5	5	3	0.555 (0.553–0.557)	62.50 (24.49–91.48)	99.98 (99.95–99.99)	50.00 (26.35–73.65)	99.99 (99.97–100.00)
*CYP2C9*											
diclofenac_paroxetine	51	9	14	4	5	10	0.347 (0.345–0.348)	28.57 (8.39–58.10)	99.98 (99.95–99.99)	44.44 (19.32–72.77)	99.96 (99.95–99.97)
ibuprofen_paroxetine	22	4	6	1	3	5	0.200 (0.198–0.202)	16.67 (0.42–64.12)	99.99 (99.97–100.00)	25.00 (3.86–73.47)	99.98 (99.97–99.99)
naproxen_paroxetine	20	6	4	3	3	1	0.600 (0.598–0.602)	75.00 (19.41–99.37)	99.99 (99.97–100.00)	50.00 (22.01–77.99)	100.00 (99.98–100.00)
diclofenac_fluoxetine	14	4	3	1	3	2	0.286 (0.283–0.289)	33.33 (0.84–90.57)	99.99 (99.97–100.00)	25.00 (4.48–70.29)	99.99 (99.98–100.00)
diclofenac_fluvoxamine	8	2	3	1	1	2	0.400 (0.397–0.403)	33.33 (0.84–90.57)	100.00 (99.98–100.00)	50.00 (7.38–92.62)	99.99 (99.98–100.00)

^a^N, the number of participants with DDIs in the Lifelines cohort (n = 80,837); ^b^N1, the number of participants with DDIs in the Lifelines cohort within overlapping population between the Lifelines and IADB.nl databases (n = 25,387); ^c^N2, the number of participants with DDIs in the IADB.nl cohort within overlapping population between the Lifelines and IADB.nl databases (n = 25,387). ^d^Over-reporting, detected only in the Lifelines cohort but not on the IADB.nl. ^e^Under-reporting, detected only in the IADB.nl but not in the Lifelines cohort.

**Figure 3:**
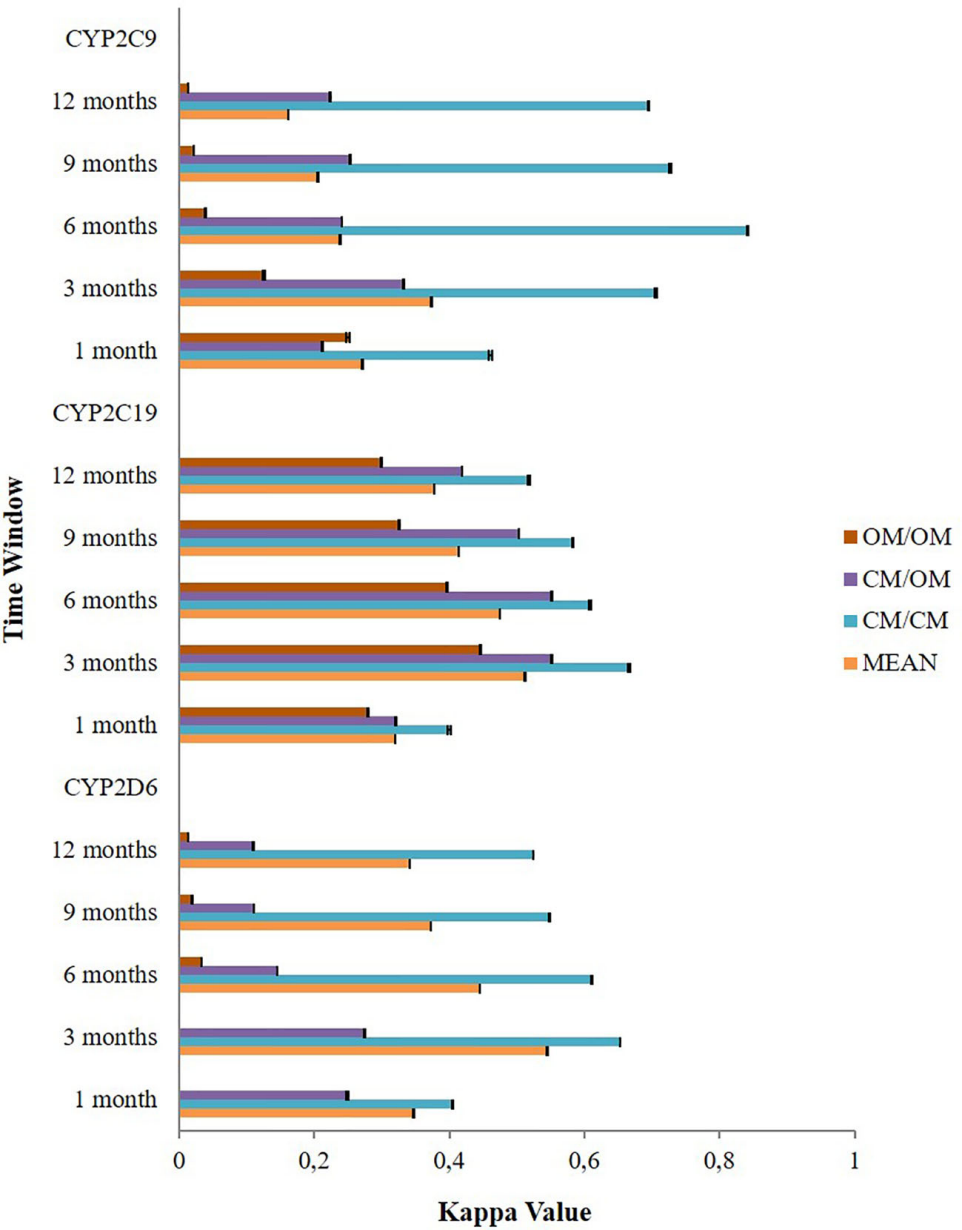
The effect of different time windows on the agreement between the Lifelines cohort and the IADB.nl database.

The application of different time windows resulted in different agreement levels of the potential DDIs (**Figure 3**). Overall, the time window of three months produced the highest mean kappa values among potential CYP2D6/2C19/2C9-mediated DDIs i.e. moderate [0.545 (95% CI: 0.544–0.545)], moderate [0.512 (95% CI: 0.511–0.512)], and fair [0.374 (95% CI: 0.373–0.375)], respectively. Extension of the time windows to 6, 9, and 12 months decreased the mean kappa values. The time window of 1 month also produced a low kappa value. For the time window of 3 months, subgroup analysis for the type of medication indicated the potential DDIs in CM-CM had better level of agreements (good) than CM-OM (fair to moderate) and OM-OM (poor to moderate). For the CYP2D6 and CYP2C9 mediated DDIs, CM-OM combination had better kappa values (fair agreement) than OM-OM combination (poor agreement). Meanwhile, for the CYP2C19 mediated DDIs, both the CM-OM and OM-OM combination had comparable agreement level (moderate). The summary of the results can be found in **Supplementary Material 3**.

**Figure 4:**
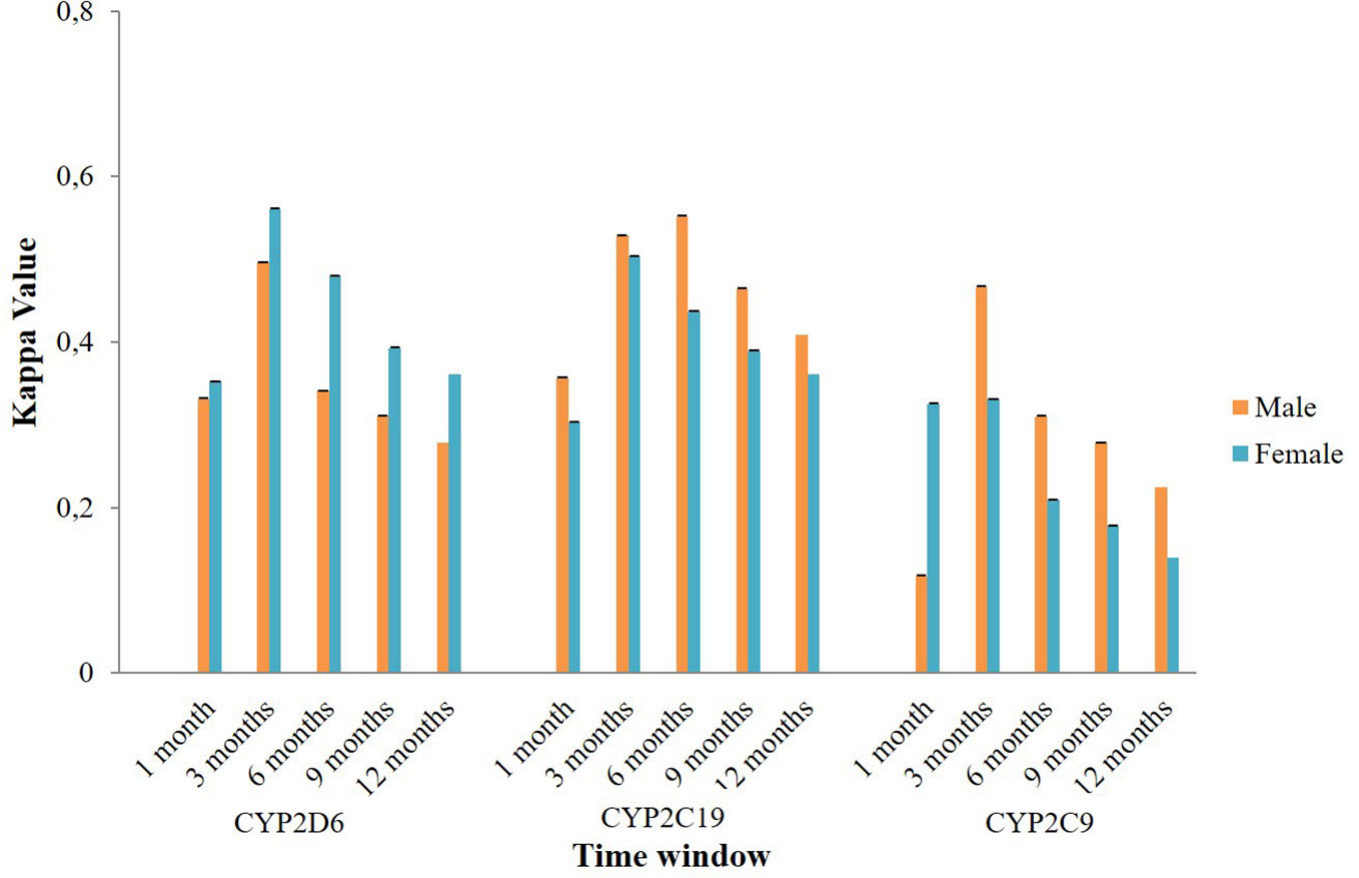
The effect of sex on the agreement between the Lifelines cohort and the IADB.nl database.

**Figure 5:**
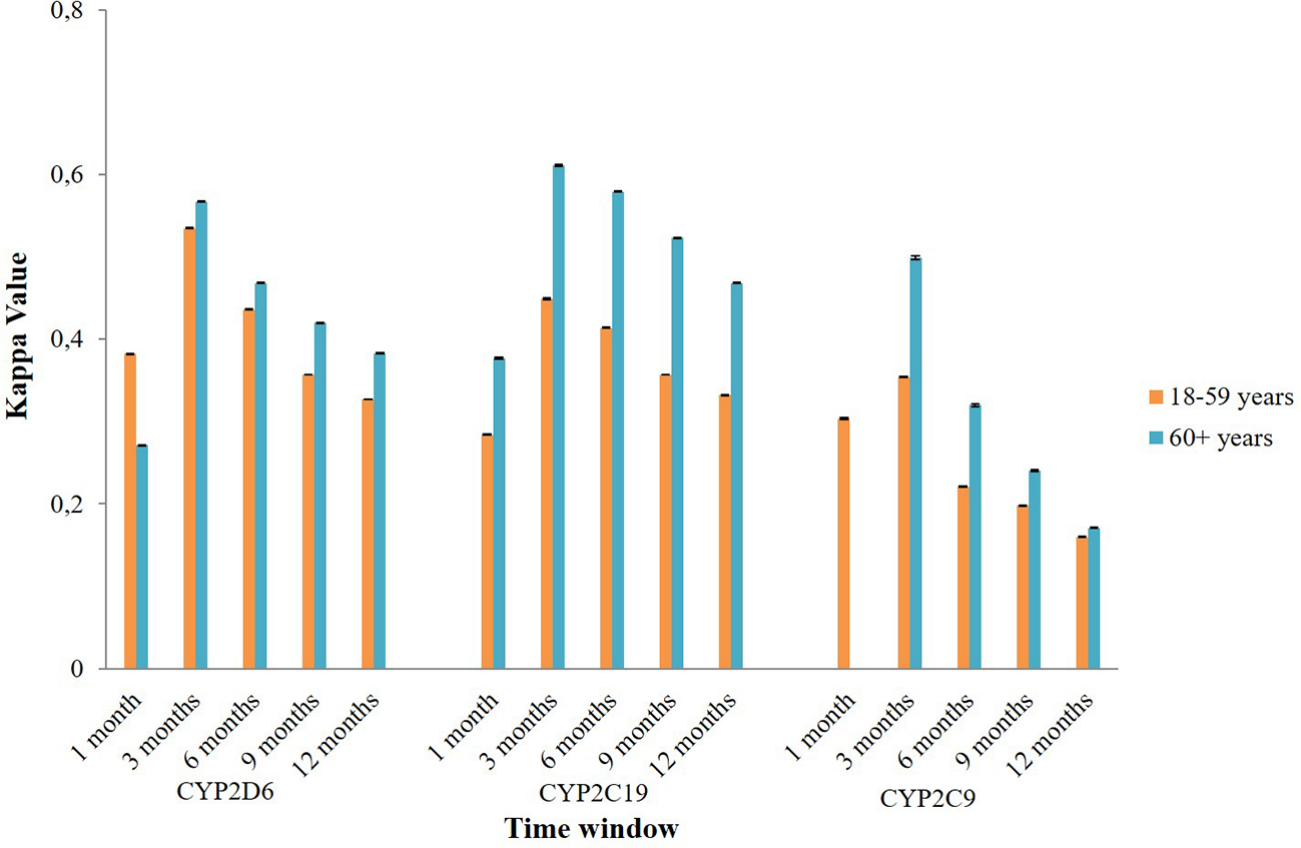
The effect of age on the agreement between the Lifelines cohort and the IADB.nl database.

Subgroup analysis of agreement by sex showed mixed results (**Figure 4**). In CYP2D6 mediated potential DDIs, females appeared to have a better level of agreement than males. The opposite result was observed in CYP2C19 and CYP2C9 mediated potential DDIs where males mostly had a better kappa value compared to females. Stratification by age indicated that people aged 60 years or older had a generally better kappa value than the younger population in CYP2D6/2C19/2C9 mediated potential DDIs (**Figure 5**).

## Discussion

In this cross-sectional study, CYP2D6/2C19/2C9-mediated potential DDIs were frequent and concordance of data varied by time window, type of medication, sex, and age. We found that one to two per hundred drug users in the Lifelines cohort were exposed to a potential CYP2D6/2C19/2C9-mediated DDI at a short moment in life time. Some of these potential DDIs are regarded as clinically relevant DDIs such as metoprolol and CYP2D6 inhibitors combinations. The DDIs may lead to bradycardia, hypotension, and atrioventricular block (Walley et al., 1993; Konig et al., 1996; Onalan et al., 2008; Bahar et al., 2018a). Other relevant DDIs were the combination of CYP2C9 inhibitors that consist of selective serotonin inhibitors (SSRIs), and nonsteroidal anti-inflammatory drugs (NSAIDs). The combination of SSRIs and NSAIDs was reported to increase risk of gastrointestinal bleedings (de Abajo et al., 1999; De Jong et al., 2003). Yet, the interaction between SSRIs and NSAIDs might be not solely a pharmacokinetic interaction but also involves a pharmacodynamic interaction (Moore et al., 2015). Our findings on the burden of DDIs might have potential clinical as well as economic implications. A DDI is one of the main contributors of an adverse drug reaction (ADR) which is one of the leading causes of hospitalisation and it can cost at minimum around €200 to €9,000 per hospitalisation (Formica et al., 2018).

Older women tended to have a higher risk to be exposed to potential CYP2D6/2C19/2C9-mediated DDIs than older men. A survey study from the United States about the pattern of drug use among adults in the outpatient setting indicated that elderly women (≥65 years old) had the highest burden of medication use in which 23% and 12% of them used at least five and 10 drugs, respectively (Kaufman et al., 2002). The risk of experiencing DDI is increased as the number of drugs taken also increased (Åstrand et al., 2006). Taking five to seven drugs and 10 to 14 drugs enhanced the risk of potentially relevant DDI by about 20%–30% and 40%–60%, respectively (Johnell and Klarin, 2007). Other studies also reported that being women and old are risk factors associated with DDIs (Grattagliano et al., 2010; Magro et al., 2012).

Based on this study, a three-month time window appeared to result in the best agreement level. This is consistent with the previous study by Sediq et al. about the validation of single drug used in the Lifelines (Sediq et al., 2018). Additionally, Lau et al. also found similar finding in their work on the validation of pharmacy records in Amsterdam, Netherlands (Lau et al., 1997). One of the possible reasons for this finding is that the Dutch reimbursement system only allows drugs to be prescribed for a maximum of 3 months period of supply (Lau et al., 1997). A comparable study to validate self-reported medication using a national prescription database in the Danish population also found a fixed 3 month time window was suitable for checking the agreement between the two sources of information on medicine use (Nielsen et al., 2008). Considering the time window is an important aspect, because a long time window may hamper the analysis of drugs used on as needed basis and a short time window may impair the analysis of drugs used chronically (Johnson and Vollmer, 1991; Nielsen et al., 2008).

Sub-analysis by type of medication indicates self-reported information on the CM-CM combination was more reliable than information on CM-OM and OM-OM combination. It offers the possibility to use co-prescription data of the CM-CM combination from the Lifelines cohort in research. This may because routinely used medication is more easily remembered by patients than drugs used occasionally. These results are consistent with previous studies (Nielsen et al., 2008; Sarangarm et al., 2012; Cohen et al., 2018).

Furthermore, for CYP2D6 and CYP2C9 related co-prescriptions, the kappa value of the OM-CM combination (fair agreement) was higher than OM-OM (poor agreement) except for CYP2C19. For the latest, the agreement level of CYP2C19 mediated OM-CM seems comparable with those of the OM-OM combination (moderate agreement). There are some possible explanations for this finding. One is the inclusion of proton pump inhibitors (PPIs) in the OM groups which are the main drugs in this group. PPIs have wide therapeutic indications and some of these indications need a chronic use of PPIs such as Zollinger-Ellisone syndrome, Barrett’s esophagus, and esophagitis (KNMP, 2018b). Another explanation is the inclusion of diazepam in the OM groups. Diazepam may be used chronically for treating patients with panic disorder and generalized anxiety disorder (KNMP, 2018a). Consequently, OM groups not solely consisted of drugs used ‘as needed’ but also may include chronically used drugs.

We found that the effect of sex on agreement is not consistent. Previous studies also reported mixed results. Some reports showed that men had a better recall accuracy than women (Linet et al., 1989; Haapea et al., 2010). Meanwhile, other studies indicated that sex had no influence on the agreement between self-reported medication use and prescription database (Van den Brandt et al., 1991; West et al., 1995). Therefore, more research is needed to determine the effect of sex on the recall accuracy, and concordance between self-reported medication use and information from a prescription database.

Our study found the agreement between the Lifelines and the IADB.nl database records is better in the older population (aged 60 years and older) than in younger adults. This result is in contrast to previous reports which found aging led to a low agreement between self-reported medication use and information from a drug database (Van den Brandt et al., 1991; West et al., 1995). A decrease in cognitive function and polypharmacy may cause poor recall information by old patients (Van den Brandt et al., 1991). However, other studies reported that age did not influence the agreement level (Sjahid et al., 1998; Lamiae et al., 2010). The method used to collect drug information may determine the influence of aging in recall bias. If the interviewers visit the patient’s house to ascertain the consumed drugs or if the patients are helped by their family in completing the questionnaire, the impact of self-reporting bias in the old participants (≥ 60 years old) can be reduced (Johnson and Vollmer, 1991; Lau et al., 1997; Richardson et al., 2013). In the Lifelines cohort, some participants filled out the questionnaire at home before visiting the premises. Therefore, the participants were potentially assisted by their relatives or may directly check their medication while completing the questionnaire. Meanwhile, some patients brought their medication at the time of interview so that interviewers could ascertain their medication list in the questionnaire.

Another possible culprit of conflicting reports is the type of medication. Most of the drugs related to CYP2D6/219/2C9 are mainly groups of drugs used by the old population chronically and were reported to be associated with a good recall such as cardiac therapy, antidiabetic agents, anti-thrombotic drugs, anticancer agents, antidepressant, and antipsychotic agents (Van den Brandt et al., 1991; Haukka et al., 2007; Gupta et al., 2011; Hafferty et al., 2018; Sediq et al., 2018). For the last two agents, Haukka et al. reported a good recall because patients brought their medication at the time of interview (Haukka et al., 2007). Lastly, the other possible explanation was the differential distribution of the population in each subgroup of age which may give a wrong impression about the influence of different age in the agreement (West et al., 1997). In our study, about 80% of the population is in the 18–59 years old subgroup. Therefore, a larger pharmaco-epidemiological study with a sub-group analysis is needed to elucidate the impact of age in the concordance of self-reported medication use and data from a prescription database.

Some strengths of our study are worth to be mentioned. Firstly, the linkage process between both databases is reliable since it was performed by CBS on individual level. Secondly, the population in our cohort is large and not limited to a certain group of population with diseases or using specific medications. Some other studies were conducted by using a limited sample and only in some particular groups of patients such as in elderly, pregnant women, patients with specific medical conditions or using certain drugs (West et al., 1995; Sjahid et al., 1998; Rockenbauer et al., 2001; Richardson et al., 2013). Thirdly, we included all types of drugs which may potentially trigger CYP2D6/219/2C9-mediated DDIs. However, there are also some limitations from our study. Firstly, we only checked the agreement of prescribed medication but not OTC drugs since the IADB.nl database has no information on OTC drugs. For example, ibuprofen is also available over the counter which may explain lower kappa values in potential DDI combinations. Next, we only had drug information from community pharmacies and, therefore, if the drugs recorded in the self-reported questionnaire were obtained from a hospital, it will not be detected in the IADB.nl and will be categorized as over-reporting information. Further, some potential DDIs included in our study were recommended to manage either by dose adjustments or monitoring of the possible potential side effects which have been possibly done by the responsible clinicians. Meanwhile, some other potential DDIs might not produce important side effects and only need caution on their use. However, we still kept them in our analysis because the influence of genetic polymorphisms on CYP2D6/2C19/2C9 may enhance the magnitude of the clinical impact of those interactions (Bahar et al., 2017a). Therefore, it would be valuable to research the interaction of CYP2D6/2C19/2C9 polymorphisms and CYP2D6/2C19/2C9-mediated DDIs in the next study. Additionally, we did not include the combination of substrates and inducers since the prevalence was too low to allow further analysis. Next, we only limited the focus of our study on the contribution of the three main phase I drug metabolizing enzymes (CYP2D6/2C19/2C9) since they cumulatively metabolize about 42% of drugs currently used in the clinical practice and mounting evidence has shown that clinical consequences of genetic polymorphisms are different among the CYP450 subfamily with CYP2D6/2C19/2C9 polymorphisms reported to have the most important clinically relevant implications (Zanger et al., 2008; Zanger and Schwab, 2013; Bahar et al., 2017a). Therefore, we assume that the CYP2D6/2C19/2C9 mediated DDIs will be the most frequent and relevant DDIs which will be found to be modified by genetic polymorphism in clinical practice. However, we would like to emphasize that drug interactions might also be facilitated by phase II drug metabolizing enzymes and drug transporters which are also subject to genetic polymorphisms and still are not optimally investigated (Board et al., 1998; Kerb, 2006). Furthermore, we only limited the analysis of the potential DDIs to the pairwise combination of medications since it reflects current guidelines and practice related to the management of DDIs in the Netherlands (van Roon et al., 2005; Heringa et al., 2016). However, the DDI might occur not only in the form of bimodal (involving two drugs) interaction but also in multimodal (involving more than two drugs) interactions especially in drugs metabolized by multiple metabolic pathways (Grönlund et al., 2011). Multimodal drug interactions were reported to produce more severe outcomes than bimodal interaction since all the metabolic pathways of the drugs are impaired (Niemi et al., 2003; Niemi et al., 2006). Lastly, our study had no clinical outcomes of the potential DDIs since in the current study our focus was limited to study the prevalence of the potential DDIs and the agreement of drug information between both databases. This study is pivotal in order to design valid follow-up studies with the aim to determine the clinical impact of the observed potential DDIs especially for chronically used medications.

## Conclusion

In conclusion, CYP2D6/2C19/2C9-mediated potential DDIs were frequent and the agreement between the Lifelines cohort and the IADB.nl differed between time windows. The best concordance level was achieved at a 3-month time window. CM-CM co-prescription had a better agreement than CM-OM and OM-OM combinations. Sex had no consistent influence on the discordance between the databases. Meanwhile, the older population had a better kappa value than the younger population. For the next drug study, the self-reporting data should be complemented with the pharmacy data in order to achieve a better accuracy in capturing the real word information on medication use.

## Data Availability Statement

The datasets for this manuscript are not publicly available, and used under license for the current study, in order to protect information that could potentially compromise the privacy of research participants. Requests to access the datasets should be directed to the Pharmlines Initiatives [email: research@lifelines.nl]. Some data such as frequency, type and agreements (kappa values) of drug-drug-interactions are available as **Supplementary Materials**.

## Ethics Statement

Ethics approval was not needed according to institutional guidelines and national legislation, since the data generated in this manuscript relied exclusively on the research database with pseudonymized information and informed consent was obtained at the time of original data collection

## Author Contributions

Conceptualization and design (MB, JB, SB, BW, EH), data curation (MB, JB), investigation (MB, JB), resources (JB, AD, RA), statistical analysis (MB), interpretation of data (MB, SB, AD, RA, BW, EH), drafting manuscript (MB), critical evaluation of the manuscript, editing, and supervision (JB, SB, AD, RA, BW, EH). All authors read and agreed on the final version of the manuscript.

## Funding

Lifelines is financially supported by several parties such as the Dutch Government, The Netherlands Organization of Scientific Research NWO (grant 175.010.2007.006), the European fund for regional development, Dutch Ministry of Economic Affairs, the Northern Netherlands Collaboration of Provinces (SNN), Provinces of Groningen and Drenthe, Pieken in de Delta, University Medical Center Groningen, and University of Groningen, Netherlands. Meanwhile, the prescription database IADB.nl and the PharmLines Initiative are financially supported by the Groningen Research Institute of Pharmacy, University of Groningen. MB obtained a DIKTI scholarship from the Ministry of Research, Technology and Higher Education of Indonesia. The funding organizations had no role and influence in the study design and results.

## Conflict of Interest

The authors declare that the research was conducted in the absence of any commercial or financial relationships that could be construed as a potential conflict of interest.
