# Supplementary material for: Prevalence and Accuracy of Information on CYP2D6, CYP2C19, and CYP2C9 Related Substrate and Inhibitor Co-Prescriptions in the General Population: A Cross‐Sectional Descriptive Study as Part of the PharmLines Initiative
Source: Front Pharmacol. 2020 May 8;11:624. doi: 10.3389/fphar.2020.00624 (PMC7225338; doi:10.3389/fphar.2020.00624)
Supplement: Supplementary file 1 [file DataSheet_1.docx]

**CYP2C19**

*Inhibitors*

CM:

Isoniazid (J04AC01), Felbamate (N03AX10), Topiramate (N03AX11), Fluoxetine (N06AB03), Fluvoxamine (N06AB08), Oral contraceptives (G03A)

OM:

Cimetidine (A02BA01), Omeprazole (A02BC01), Pantoprazole (A02BC02), Esomeprazole (A02BC05), Lansoprazole (A02BD07), Voriconazole (J02AC03), Ketoconazole (J02AB02)

*Substrate*

CM:

Labetalol (C07AG01), amitriptyline (N06AA09), citalopram (N06AB04), clomipramine (N06AA04), clopidogrel (B01AC04), cyclophosphamide (L01AA01), imipramine (N06AA02)

OM:

Esomeprazole (A02BC05), lansoprazole (A02BC03), omeprazole (A02BC01), pantoprazole (A02BC02), diazepam (N05BA01), phenobarbital (N03AA02), proguanil (P01BB01), voriconazole (J02AC03),

**CYP2C9**

*Inhibitor*

CM:

Amiodarone (C01BD01), Fluvoxamine (N06AB08), Efavirenz (J05AG03), Isoniazid (J04AC01), Paroxetine (N06AB05), Fluoxetine (N06AB03), Teniposide (L01CB02)

OM

Fluconazole (J02AC01), Voriconazole (J02AC03), Metronidazole (P01AB01), Phenylbutazone (M01AA01), Sulfamethoxazole (J01EC01), Benzbromaron (M04AB03), Cimetidine (A02BA01), Cotrimoxazole (J01EE01), Miconazole (A07AC01)

*Substrate*

CM:

Tolbutamide (A10BB03), Glipizide (A10BB07), Losartan (C09CA01), Irbesartan (C09CA04), Glibenclamide (A10BB01), Glimepiride (A10BB12), Fluvastatin (C10AA04), Phenytoin (N03AB02), Rosiglitazone (A10BG02), Valproic Acid (N03AG01), S-warfarin (B01AA03), Zafirlukast (R03DC01)

OM:

Diclofenac (M01AB05), Ibuprofen (M01AE01), Naproxen (M01AE02), Piroxicam (M01AC01), Celecoxib (M01AH01)

**CYP2D6**

*Inhibitor*

CM:

Propafenon (C01BC03), Amiodarone (C01BD01), Mirabegron (G04BD12), Cinacalcet (H05BX01), Panobinostat (L01XX42), Abirateron (L02BX03), Haloperidol (N05AD01), Aripiprazole (N05AX12), Clomipramine (N06AA04), Doxepin (N06AA12), Paroxetine (N06AB05), Sertraline (N06AB06), Bupropion (N06AX12), Venlafaxine (N06AX16), Duloxetine (N06AX21), Methadone (N07BC02), Fluoxetine (N06AB03), Fluvoxamine (N06AB08), Ritonavir (J05AE03), Terbinafin (D01BA02), Chlorpromazine (N05AA01), Moclobemide (N06AG02).

OM:

Cimetidine (A02BA01), Dexchlorpheniramine (R06AB02), Diphenhydramine (R06AA02), Metoclopramide (A03FA01).

*Substrate*

CM:

Carvedilol (C07AG02), S-metoprolol (C07AB02), Propafenone ( C01BC03), Timolol (C07AA06), Amitriptyline (N06AA09), Clomipramine (N06AA04), Desipramine (N06AA01), Duloxetine (N06AX21), Fluoxetine (N06AB03), Imipramine (N06AA02), Paroxetine (N06AB05), Nortriptyline (N06AA10), Venlafaxine (N06AX16), Haloperidol (N05AD01), Risperidone (N05AX08), Thioridazine (N05AC02), Aripiprazole (N05AX12), Atomoxetine (N06BA09), Doxepine (N06AA12), Flecainide (C01BC04), Mexiletine (C01BB02), Tamoxifen (L02BA01).

OM:

Codeine (R05DA04), Dextromethorphan (R05DA09), Ondansetron (A04AA01), Oxycodone (N02AA05), Tramadol (N02AX02).
