# Supplementary material for: Prevalence and Accuracy of Information on CYP2D6, CYP2C19, and CYP2C9 Related Substrate and Inhibitor Co-Prescriptions in the General Population: A Cross‐Sectional Descriptive Study as Part of the PharmLines Initiative
Source: Front Pharmacol. 2020 May 8;11:624. doi: 10.3389/fphar.2020.00624 (PMC7225338; doi:10.3389/fphar.2020.00624)
Supplement: Supplementary file 2 [file DataSheet_2.docx]

The number of patients with potential DDIs in the Lifelines cohort

Classification of potential DDIs based on the recommended management provided by Epocrates^®^:

- Contraindicated: life threatening and permanent damage may be induced = 4,
- Avoid combination/use alternative: can cause therapeutic problems/ difficulties but may be administered together if the patient is carefully monitored = 3,
- modify treatment/monitor: cause increased or reduced effects but to a lesser extent, effects are mainly expressed in already chronic disease compromised patients = 2,
- Caution: caution on use, mainly cause unimportant effects and no specific action is required = 1,
- No record = 0.

CYP2D6

| *Total* |  | Epocrates^®^ | Drugs.com |
| --- | --- | --- | --- |
| metoprolol_paroxetine | 83 | 2 |  |
| metoprolol_clomipramine | 18 | 2 |  |
| metoprolol_fluoxetine | 17 | 2 |  |
| metoprolol_amiodarone | 14 | 2 |  |
| metoprolol_duloxetine | 14 | 2 |  |
| risperidone_venlafaxine | 13 | 1 |  |
| tramadol_venlafaxine | 13 | 3 |  |
| metoprolol_sertraline | 12 | 1 |  |
| tramadol_paroxetine | 12 | 3 |  |
| amitriptyline_venlafaxine | 11 | 2 |  |
| tramadol_metocloperamide | 10 | 2 |  |
| metoprolol_fluovoxamine | 9 | 0 | 2 |
| metoprolol_terbinafine | 9 | 2 |  |
| tamoxifen_venlafaxine | 9 | 0 | 2 |
| amitriptyline_paroxetine | 8 | 2 |  |
| tramadol_duloxetine | 8 | 3 |  |
| amitriptyline_fluoxetine | 7 | 3 |  |
| amitriptyline_metocloperamide | 7 | 3 |  |
| flecainide_paroxetine | 7 | 2 |  |
| oxycodone_duloxetine | 7 | 3 |  |
| tramadol_fluoxetine | 7 | 3 |  |
| venlafaxine_paroxetine | 6 | 3 |  |
| venlafaxine_metocloperamide | 6 | 1 |  |
| risperidone_clomipramine | 6 | 2 |  |
| risperidone_paroxetine | 6 | 2 |  |
| risperidone_fluoxetine | 6 | 2 |  |
| amitriptyline_duloxetine | 5 | 2 |  |
| paroxetine_metocloperamide | 5 | 2 |  |
| oxycodone_paroxetine | 5 | 3 |  |
| amitriptyline_sertraline | 4 | 2 |  |
| paroxetine_aripiprazole | 4 | 2 |  |
| paroxetine_terbinafine | 4 | 2 |  |
| venlafaxine_aripirazole | 4 | 1 |  |
| venlafaxine_terbinafine | 4 | 1 |  |
| risperidone_duloxetine | 4 | 2 |  |
| metoprolol_propafenone | 3 | 2 |  |
| metoprolol_moclobemide | 3 | 2 | 0 |
| amitriptyline_haloperidol | 3 | 2 |  |
| fluoxetine_venlafaxine | 3 | 3 |  |
| paroxetine_sertraline | 3 | 3 |  |
| nortriptyline_sertraline | 3 | 2 |  |
| nortriptyline_venlafaxine | 3 | 2 |  |
| flecainide_duloxetine | 3 | 2 |  |
| oxycodone_venlafaxine | 3 | 3 |  |
| carvedilol_amiodarone | 2 | 2 |  |
| metoprolol_cimetidine | 2 | 1 |  |
| metoprolol_cinacalcet | 2 | 2 |  |
| metoprolol_haloperidol | 2 | 2 |  |
| metoprolol_aripiprazole | 2 | 2 |  |
| amitriptyline_aripiprazole | 2 | 2 |  |
| amitriptyline_dexchlorpheniramine | 2 | 0 | 1 |
| clomipramine_aripiprazole | 2 | 2 |  |
| clomipramine_fluvoxamine | 2 | 2 |  |
| duloxetine_metocloperamide | 2 | 1 |  |
| fluoxetine_haloperidol | 2 | 3 |  |
| paroxetine_duloxetine | 2 | 3 |  |
| nortriptyline_fluoxetine | 2 | 3 |  |
| risperidone_aripiprazole | 2 | 2 |  |
| risperidone_sertraline | 2 | 1 |  |
| aripiprazole_sertraline | 2 | 1 |  |
| codeine_paroxetine | 2 | 3 |  |
| codeine_dexchlorpheniramine | 2 | 0 | 2 |
| codeine_fluoxetine | 2 | 3 |  |
| flecainide_sertraline | 2 | 2 |  |
| oxycodone_methadone | 2 | 3 |  |
| oxycodone_metocloperamide | 2 | 2 |  |
| tramadol_sertraline | 2 | 3 |  |
| tramadol_methadone | 2 | 3 |  |
| tramadol_terbinafine | 2 | 3 |  |
| metoprolol_doxepine | 1 | 0 | 1 |
| metoprolol_methadone | 1 | 2 |  |
| amitriptyline_cimetidine | 1 | 0 | 2 |
| amitriptyline_amiodarone | 1 | 2 |  |
| amitriptyline_fluvoxamine | 1 | 2 |  |
| amitriptyline_terbinafine | 1 | 2 |  |
| clomipramine_paroxetine | 1 | 2 |  |
| clomipramine_terbinafine | 1 | 0 | 2 |
| clomipramine_metocloperamide | 1 | 3 |  |
| duloxetine_clomipramine | 1 | 2 |  |
| duloxetine_venlafaxine | 1 | 3 |  |
| duloxetine_terbinafine | 1 | 1 |  |
| fluoxetine_amiodarone | 1 | 3 |  |
| fluoxetine_duloxetine | 1 | 3 |  |
| fluoxetine_metocloperamide | 1 | 2 |  |
| imipramine_sertraline | 1 | 2 |  |
| paroxetine_cimetidine | 1 | 2 |  |
| paroxetine_haloperidol | 1 | 2 |  |
| paroxetine_fluovoxamine | 1 | 3 |  |
| nortriptyline_aripiprazole | 1 | 2 |  |
| nortriptyline_clomipramine | 1 | 3 |  |
| nortriptyline_doxepine | 1 | 3 |  |
| nortriptyline_paroxetine | 1 | 2 |  |
| nortriptyline_terbinafine | 1 | 2 |  |
| venlafaxine_haloperidol | 1 | 3 |  |
| venlafaxine_bupropion | 1 | 2 |  |
| venlafaxine_methadone | 1 | 3 |  |
| haloperidol_aripiprazole | 1 | 2 |  |
| haloperidol_fluvoxamine | 1 | 2 |  |
| risperidone_fluvoxamine | 1 | 1 |  |
| aripiprazole_fluoxetine | 1 | 2 |  |
| aripiprazole_fluvoxamine | 1 | 1 |  |
| atomoxetine_duloxetine | 1 | 0 | 2 |
| codeine_clomipramine | 1 | 3 |  |
| codeine_venlafaxine | 1 | 3 |  |
| codeine_duloxetine | 1 | 3 |  |
| codeine_fluvoxamine | 1 | 3 |  |
| codeine_terbinafine | 1 | 3 |  |
| flecainide_venlafaxine | 1 | 0 | 2 |
| oxycodone_amiodarone | 1 | 3 |  |
| oxycodone_haloperidol | 1 | 2 |  |
| oxycodone_sertraline | 1 | 3 |  |
| oxycodone_fluoxetine | 1 | 3 |  |
| tamoxifen_paroxetine | 1 | 3 |  |
| tramadol_cimetidine | 1 | 3 |  |
| tramadol_aripiprazole | 1 | 2 |  |
| tramadol_clomipramine | 1 | 3 |  |

CYP2C19

| *Total* |  | Epocrates^®^ | Drugs.com |
| --- | --- | --- | --- |
| citalopram_omeprazole | 173 | 2 |  |
| diazepam_omeprazole | 151 | 3 |  |
| omeprazole_fluvoxamine | 28 | 1 |  |
| diazepam_esomeprazole | 27 | 3 |  |
| clopidogrel_omeprazole | 24 | 3 |  |
| citalopram_esomeprazole | 22 | 2 |  |
| diazepam_fluoxetine | 13 | 3 |  |
| clopidogrel_esomeprazole | 10 | 3 |  |
| amitriptyline_topiramate | 8 | 1 |  |
| citalopram_topiramate | 8 | 1 |  |
| propranolol_fluoxetine | 7 | 2 |  |
| diazepam_topiramate | 5 | 2 |  |
| propranolol_fluvoxamine | 5 | 2 |  |
| esomeprazole_fluvoxamine | 4 | 1 |  |
| omeprazole_ketoconazole | 4 | 3 |  |
| citalopram_fluoxetine | 4 | 3 |  |
| diazepam_fluvoxamine | 3 | 3 |  |
| labetalol_fluoxetine | 2 | 0 | 2 |
| clomipramine_esomeprazole | 2 | 0 | 1 |
| clomipramine_fluvoxamine | 2 | 2 |  |
| clopidogrel_fluoxetine | 2 | 3 |  |
| lansoprazole_fluvoxamine | 1 | 1 |  |
| diazepam_contraceptives | 1 | 1 | 0 |
| phenobarbital_omeprazole | 1 | 1 |  |
| amitriptyline_cimetidine | 1 | 0 | 2 |
| amitriptyline_fluvoxamine | 1 | 2 |  |
| citalopram_cimetidine | 1 | 2 |  |
| citalopram_fluvoxamine | 1 | 3 |  |
| clomipramine_topiramate | 1 | 1 |  |
| clopidogrel_lansoprazole | 1 | 0 | 2 |

CYP2C9

| *Total* |  | Epocrates^®^ | Drugs.com |
| --- | --- | --- | --- |
| diclofenac_paroxetine | 51 | 2 |  |
| ibuprofen_paroxetine | 22 | 2 |  |
| naproxen_paroxetine | 20 | 2 |  |
| diclofenac_fluoxetine | 14 | 2 |  |
| diclofenac_fluvoxamine | 8 | 2 |  |
| valproic acid_paroxetine | 8 | 2 |  |
| diclofenac_fluconazole | 5 | 2 |  |
| meloxicam_paroxetine | 5 | 2 |  |
| naproxen_fluoxetine | 5 | 2 |  |
| losartan_fluvoxamine | 5 | 0 | 1 |
| glimepiride_paroxetine | 4 | 2 |  |
| valproic acid_fluoxetine | 4 | 2 |  |
| diclofenac_metronidazole | 3 | 2 |  |
| diclofenac_cimetidine | 3 | 0 | 1 |
| ibuprofen_fluvoxamine | 3 | 2 |  |
| tolbutamide_paroxetine | 3 | 2 |  |
| tolbutamide_fluoxetine | 3 | 2 |  |
| diclofenac_cotrimoxazole | 2 | 2 |  |
| diclofenac_miconazole | 2 | 0 | 1 |
| ibuprofen_cotrimoxazole | 2 | 2 |  |
| ibuprofen_fluoxetine | 9 | 2 |  |
| meloxicam_fluoxetine | 2 | 2 |  |
| naproxen_fluvoxamine | 2 | 2 |  |
| ibuprofen_metronidazole | 1 | 1 | 0 |
| meloxicam_fluconazole | 1 | 0 | 2 |
| naproxen_metronidazole | 1 | 1 | 0 |
| naproxen_cotrimoxazole | 1 | 2 |  |
| tolbutamide_amiodarone | 1 | 2 |  |
| tolbutamide_cimetidine | 1 | 0 | 2 |
| tolbutamide_cotrimoxazole | 1 | 2 |  |
| losartan_fluconazole | 1 | 0 | 2 |
| fluoxetine_fluconazole | 1 | 0 | 2 |
| fluvastatin_efavirenz | 1 | 0 | 2 |
| valproic acid_fluvoxamine | 1 | 2 |  |
| valproic acid_metronidazole | 1 | 1 |  |
| valproic acid_cimetidine | 1 | 2 |  |
