# Supplementary material for: Prevalence and Accuracy of Information on CYP2D6, CYP2C19, and CYP2C9 Related Substrate and Inhibitor Co-Prescriptions in the General Population: A Cross‐Sectional Descriptive Study as Part of the PharmLines Initiative
Source: Front Pharmacol. 2020 May 8;11:624. doi: 10.3389/fphar.2020.00624 (PMC7225338; doi:10.3389/fphar.2020.00624)
Supplement: Supplementary file 3 [file DataSheet_3.docx]

Altman et al. provided some guidelines to define the Cohen’s kappa values i.e. poor (<0.20), fair (0.20-0.40), moderate (0.41-0.60), good (0.61-0.80), and very good (0.81-1.00) (Altman 1990).

Summary of the results

**CYP2D6**

Kappa value after being stratified by type of medication.

| Time Window | Overall | | CM_CM combination | | CM_OM combination | | OM_OM combination | |
| --- | --- | --- | --- | --- | --- | --- | --- | --- |
|  | Kappa | SD | Kappa | SD | Kappa | SD | Kappa | SD |
| 1 month | 0.347 | 0.037 | 0.405 | 0.043 | 0.249 | 0.083 | 0.000 | 0.000 |
| 3 months | 0.545 | 0.030 | 0.653 | 0.033 | 0.275 | 0.063 | 0.000 | 0.000 |
| 6 months | 0.445 | 0.027 | 0.611 | 0.031 | 0.145 | 0.037 | 0.033 | 0.032 |
| 9 months | 0.373 | 0.024 | 0.548 | 0.030 | 0.111 | 0.029 | 0.019 | 0.018 |
| 12 months | 0.341 | 0.023 | 0.524 | 0.030 | 0.110 | 0.027 | 0.014 | 0.014 |

Kappa value after being stratified by type of gender.

| Time Window | Male | | Female | |
| --- | --- | --- | --- | --- |
|  | Kappa | SD | Kappa | SD |
| 1 month | 0.331 | 0.083 | 0.352 | 0.041 |
| 3 months | 0.496 | 0.063 | 0.561 | 0.035 |
| 6 months | 0.341 | 0.052 | 0.480 | 0.031 |
| 9 months | 0.311 | 0.048 | 0.393 | 0.028 |
| 12 months | 0.278 | 0.044 | 0.361 | 0.026 |

Kappa value after being stratified by type of age.

| Time Window | 18-59 years old | | 60+ years old | |
| --- | --- | --- | --- | --- |
|  | Kappa | SD | Kappa | SD |
| 1 month | 0.382 | 0.046 | 0.271 | 0.056 |
| 3 months | 0.535 | 0.037 | 0.567 | 0.052 |
| 6 months | 0.436 | 0.032 | 0.468 | 0.048 |
| 9 months | 0.357 | 0.029 | 0.420 | 0.045 |
| 12 months | 0.327 | 0.027 | 0.383 | 0.043 |

**CYP2C19**

Kappa value after being stratified by type of medication.

| Time Window | Overall | | Chronic_Chronic Medication | | Chronic _Acute Medication | | Acute_Acute Medication | |
| --- | --- | --- | --- | --- | --- | --- | --- | --- |
|  | Kappa | SD | Kappa | SD | Kappa | SD | Kappa | SD |
| 1 month | 0.320 | 0.047 | 0.400 | 0.160 | 0.321 | 0.063 | 0.280 | 0.074 |
| 3 months | 0.512 | 0.039 | 0.666 | 0.119 | 0.551 | 0.054 | 0.446 | 0.060 |
| 6 months | 0.475 | 0.035 | 0.608 | 0.120 | 0.551 | 0.049 | 0.397 | 0.050 |
| 9 months | 0.414 | 0.032 | 0.583 | 0.120 | 0.503 | 0.047 | 0.326 | 0.044 |
| 12 months | 0.378 | 0.030 | 0.518 | 0.117 | 0.419 | 0.044 | 0.299 | 0.041 |

Kappa value after being stratified by type of gender.

| Time Window | Male | | Female | |
| --- | --- | --- | --- | --- |
|  | Kappa | SD | Kappa | SD |
| 1 month | 0.356 | 0.082 | 0.303 | 0.057 |
| 3 months | 0.529 | 0.070 | 0.504 | 0.047 |
| 6 months | 0.552 | 0.059 | 0.437 | 0.043 |
| 9 months | 0.464 | 0.056 | 0.389 | 0.040 |
| 12 months | 0.409 | 0.051 | 0.361 | 0.038 |

Kappa value after being stratified by type of age.

| Time Window | 18-59 years old | | 60+ years old | |
| --- | --- | --- | --- | --- |
|  | Kappa | SD | Kappa | SD |
| 1 month | 0.284 | 0.058 | 0.377 | 0.078 |
| 3 months | 0.449 | 0.051 | 0.611 | 0.060 |
| 6 months | 0.414 | 0.044 | 0.579 | 0.055 |
| 9 months | 0.357 | 0.040 | 0.523 | 0.054 |
| 12 months | 0.332 | 0.037 | 0.468 | 0.052 |

**CYP2C9**

Kappa value after being stratified by type of medication.

| Time Window | Overall | | CM_CM combination | | CM_OM combination | | OM_OM combination | |
| --- | --- | --- | --- | --- | --- | --- | --- | --- |
|  | Kappa | SD | Kappa | SD | Kappa | SD | Kappa | SD |
| 1 month | 0.272 | 0.072 | 0.461 | 0.171 | 0.212 | 0.080 | 0.250 | 0.203 |
| 3 months | 0.374 | 0.060 | 0.706 | 0.126 | 0.332 | 0.072 | 0.125 | 0.113 |
| 6 months | 0.238 | 0.042 | 0.842 | 0.090 | 0.241 | 0.055 | 0.039 | 0.039 |
| 9 months | 0.205 | 0.035 | 0.727 | 0.107 | 0.253 | 0.049 | 0.022 | 0.023 |
| 12 months | 0.162 | 0.027 | 0.695 | 0.110 | 0.224 | 0.041 | 0.013 | 0.014 |

Kappa value after being stratified by type of gender.

| Time Window | Male | | Female | |
| --- | --- | --- | --- | --- |
|  | Kappa | SD | Kappa | SD |
| 1 month | 0.117 | 0.107 | 0.325 | 0.087 |
| 3 months | 0.467 | 0.106 | 0.331 | 0.072 |
| 6 months | 0.310 | 0.085 | 0.209 | 0.048 |
| 9 months | 0.278 | 0.074 | 0.178 | 0.038 |
| 12 months | 0.224 | 0.059 | 0.139 | 0.030 |

Kappa value after being stratified by type of age.

| Time Window | 18-59 years old | | 60+ years old | |
| --- | --- | --- | --- | --- |
|  | Kappa | SD | Kappa | SD |
| 1 month | 0.304 | 0.078 | 0.000 | 0.000 |
| 3 months | 0.354 | 0.065 | 0.499 | 0.152 |
| 6 months | 0.221 | 0.046 | 0.320 | 0.109 |
| 9 months | 0.198 | 0.038 | 0.241 | 0.090 |
| 12 months | 0.160 | 0.029 | 0.171 | 0.068 |
